# High-phosphorus diets reduce aortic lesions and cardiomyocyte size and modify lipid metabolism in Ldl receptor knockout mice

**DOI:** 10.1038/s41598-020-77509-w

**Published:** 2020-11-27

**Authors:** Sarah M. Grundmann, Alexandra Schutkowski, Christian Berger, Anja C. Baur, Bettina König, Gabriele I. Stangl

**Affiliations:** 1 Institute of Agricultural and Nutritional Sciences, Martin Luther University Halle-Wittenberg, Von-Danckelmann-Platz 2, 06120 Halle (Saale), Germany; 2 Competence Cluster for Nutrition and Cardiovascular Health (nutriCARD), Halle-Jena-Leipzig, Leipzig, Germany

**Keywords:** Physiology, Molecular medicine

## Abstract

The consumption of phosphorus in Western populations largely exceeds the recommended intake, while vitamin D supply is often insufficient. Both situations are linked to an increased cardiovascular risk. A 17-week two-factorial study with Ldl receptor^-/-^ mice was conducted to investigate the cardiovascular impact of dietary phosphorus [adequate (0.3%; P_0.3_) vs. high (1.5%; P_1.5_)] in combination with a low (50 IU/kg; D_50_) or adequate vitamin D diet (1000 IU/kg; D_1000_). The data demonstrate that mice fed the P_1.5_ vs. P_0.3_ diets developed smaller vascular lesions (*p* = 0.013) and cardiac hypotrophy (*p* = 0.011), which were accompanied by diminished IGF1 and insulin signalling activity in their hearts. Vitamin D showed no independent effect on atherogenesis and heart morphology. Feeding P_1.5_ vs. P_0.3_ diets resulted in markedly reduced serum triacylglycerols (*p* < 0.0001) and cholesterol (*p* < 0.0001), higher faecal lipid excretion (*p* < 0.0001) and a reduced mRNA abundance of hepatic sterol exporters and lipoprotein receptors. Minor hypocholesterolaemic and hypotriglyceridaemic effects were also found in mice fed the D_1000_ vs. D_50_ diets (*p* = 0.048, *p* = 0.026). To conclude, a high phosphorus intake strongly affected the formation of vascular lesions, cardiac morphology, and lipid metabolism, although these changes are not indicative of an increased cardiovascular risk.

## Introduction

Phosphorus is an essential mineral in human and animal nutrition. The vast majority of phosphorus is bound as hydroxyapatite, which, in combination with calcium, ensures the stability of bone^1^. It is further a component of cell membrane phospholipids, forms the backbone of DNA and RNA (along with sugars), regulates blood pH and is involved in energy-driven cell processes (e.g., as a component of ATP or GTP) and intracellular signalling^2^. It is suggested that dietary phosphorus intake in populations consuming Western diets largely exceeds the recommended intake^3^. Phosphorus is primarily present in food in the form of phosphate. The main sources of phosphate are foods rich in protein, such as dairy and meat products, as well as inorganic phosphate additives that are commonly used to produce processed food. In contrast to natural food, phosphorus from food additives is suggested to be absorbed in large quantities, as shown by the high postprandial serum levels of phosphate after the intake of phosphate-containing food additives^4,5^.

Excessive consumption of phosphorus has been associated with detrimental effects on health. Animal studies have consistently shown that high-phosphorus diets can reduce bone mass^6,7^. Another disease spectrum that has been linked to a high phosphorus intake is cardiovascular disease (CVD). Most of the data come from studies with patients suffering from chronic kidney disease (CKD). CKD is characterised by the inability of the kidney to eliminate an excess of phosphorus, which in turn results in endothelial dysfunction and vascular calcification^8–10^. However, the role of excessive dietary phosphorus in the development of CVD in individuals with normal kidney function is being discussed. Epidemiological studies that investigated the associations between phosphorus intake/serum phosphorus and CVDs did not show consistent and conclusive results. Data obtained from the Framingham Offspring cohort show that higher serum phosphorus concentrations were associated with an increased CVD risk in individuals free of CKD and CVD^11^. In a cross-sectional epidemiologic study, increased phosphorus intake was associated with a reduction in blood pressure^12^, and in another prospective study, intake of phosphorus from dairy products but not from other food sources was linked to a reduced risk of incident hypertension^13^. Findings from the prospective Coronary Artery Risk Development in Young Adults (CARDIA) study demonstrate that higher serum phosphorus levels, even within the normal range, appear to be a risk factor for coronary artery atherosclerosis in healthy adults^14^. Additionally, data from a community-based study with individuals free of known CVD showed that dietary phosphorus intake was associated with greater left ventricular mass in women but not in men^15^. In contrast, another study that included patients with stable CVD showed that higher serum phosphorus levels are associated with a greater left ventricular mass in men, but not in women^16^. However, other epidemiological studies did not find significant associations between phosphorus intake/serum phosphorus levels and cardiovascular mortality^17^.

Apart from the fact that results from epidemiological studies do not allow any statement regarding causal relationships between dietary phosphorus and disease risk, several limitations of these studies do not allow clear conclusions concerning the role of phosphorus in CVD development and progression. One limitation is the precise assessment of dietary phosphorus intake. Available reference sources often underestimate the true phosphorus content of food, as they do not consider phosphorus that comes from food additives^18^. Another point is that it is difficult to estimate the true phosphorus intake from serum measurements as the serum phosphorus is tightly regulated^19,20^. Changes in serum phosphorus levels can be found predominantly in the postprandial state but not necessarily in the fasting state^4,5^.

Hormonal regulators of phosphorus homeostasis are bone-derived fibroblast growth factor 23 (FGF23), parathyroid hormone (PTH) and calcitriol, the biologically active form of vitamin D. FGF23 and PTH stimulate the renal excretion of phosphorus by downregulating sodium-phosphate transporters in renal proximal tubule cells^21,22^. FGF23 also reduces intestinal absorption of phosphorus by reducing calcitriol formation through the inhibition of renal 1α-hydroxylase^23^, while calcitriol mainly stimulates intestinal and renal phosphorus absorption^24,25^. Vitamin D insufficiency is widespread^26,27^, and recent meta-analyses of prospective cohort studies show that low levels of circulating vitamin D are associated with higher CVD morbidity and mortality^28,29^.

The current study aimed to investigate the role of dietary inorganic phosphorus in vascular lesion development, heart morphology and CVD risk in mice fed a diet that contained low or adequate amounts of vitamin D. Therefore, a long-term study using low-density lipoprotein receptor (Ldlr) knockout mice as a model of atherosclerosis was conducted. Fifty-six, 9-week-old male mice were randomized to four groups of fourteen animals each and fed a Western type diet with adequate (1000 IU; D_1000_) or low (50 IU; D_50_) vitamin D concentrations that contained either adequate (0.3%; P_0.3_) or high (1.5%; P_1.5_) phosphorus concentrations over a 17-week period.

## Results

### Body weights and tibia lengths

The body weights of mice were only marginally influenced by dietary phosphorus but not by vitamin D. Mice that received the P_1.5_ diets had slightly lower body weights (approximately -5%) than mice fed the P_0.3_ diets (means ± standard deviation in g: P_0.3_D_50_ 29.8 ± 1.9; P_0.3_D_1000_ 31.6 ± 3.4; P_1.5_D_50_ 29.5 ± 1.6; P_1.5_D_1000_ 28.9 ± 1.7, *p*-values: P 0.016, D 0.380, PxD 0.056). The tibia length as a marker of body size did not differ significantly between the four groups of mice (means ± standard deviation in mm: P_0.3_D_50_ 17.8 ± 0.3; P_0.3_D_1000_ 17.9 ± 0.3; P_1.5_D_50_ 18.0 ± 0.3; P_1.5_D_1000_ 18.0 ± 0.3, *p*-values: P 0.148, D 0.922, PxD 0.466).

### Mineral status and phosphorus-regulating hormones

As depicted in Table 1, the serum concentration of inorganic phosphate was neither influenced by dietary phosphorus nor by dietary vitamin D. In contrast, the serum concentrations of calcium and the phosphaturic hormones intact FGF23 (iFGF23) and intact PTH (iPTH) were significantly affected by phosphorus in the diet. The serum of mice that received the P_1.5_ diets was characterised by lower concentrations of calcium and higher concentrations of iFGF23 and iPTH than the serum of mice fed the P_0.3_ diets (Table 1). To assess the vitamin D status of mice, the serum concentrations of 25-hydroxyvitamin D (25(OH)D) and 1,25-dihydroxyvitamin D (1,25(OH)_2_D) were analysed. Statistical analysis revealed that the 25(OH)D concentration in serum was influenced by dietary vitamin D but not by phosphorus. The serum concentrations of 25(OH)D were significantly lower in the mice fed the D_50_ diets than in those fed the D_1000_ diets (Table 1). Dietary treatments also affected the serum concentration of 1,25(OH)_2_D. Here, we found that the dietary combination of P_1.5_ and D_1000_ resulted in markedly higher serum concentrations of 1,25(OH)_2_D than feeding the P_0.3_D_1000_ or P_1.5_D_50_ diets (Table 1).

**Table 1:** Vitamin D status, serum concentrations of ionic calcium and inorganic phosphate and hormones involved in the regulation of phosphorus and calcium metabolism in 26-week-old low-density lipoprotein receptor knockout (Ldlr^-/-^) mice.

Parameter	P_0.3_D_50_	P_0.3_D_1000_	P_1.5_D_50_	P_1.5_D_1000_	2-WA *p*-values		
					P	D	P × D
Inorganic phosphate in mmol/l	1.95 ± 0.19	1.99 ± 0.21	2.03 ± 0.29	1.99 ± 0.15	0.427	0.966	0.471
Ionic calcium in mmol/l	2.6 ± 0.28	2.74 ± 0.16	2.56 ± 0.12	2.61 ± 0.27	0.039	0.372	0.990
iFGF23 in pg/ml	349 ± 146	363 ± 144	2230 ± 697	2491 ± 1196	< .0001	0.608	0.923
iPTH in pg/ml	137 ± 120	112 ± 95	388 ± 301	352 ± 353	< .0001	0.320	0.941
25(OH)D in nmol/l	28.0 ± 8.1	56.6 ± 15.1	19.9 ± 3.8	58.5 ± 11.6	0.290	< .0001	0.095
1,25(OH)_2_D in pmol/l	204 ± 57	247 ± 89*	281 ± 60*	598 ± 125	< .0001	< .0001	< .0001

Mice were fed a Western diet with adequate (0.3%; P_0.3_) or high (1.5%; P_1.5_) phosphorus levels coupled with adequate (1000 IU; D_1000_) or low (50 IU; D_50_) vitamin D levels over a 17-week period.

Intact fibroblast growth factor 23 (iFGF23); intact parathyroid hormone (iPTH); Data are provided as mean ± standard deviation [n = 14]. Data were analysed via two-factorial analysis of variance (2-WA) (significance level *p* < 0.05) with the classification factors phosphorus and vitamin D as well as their interaction. *differs from P_1.5_D_1000._

### Quantification and characterization of vascular lesions

**Figure 1:**
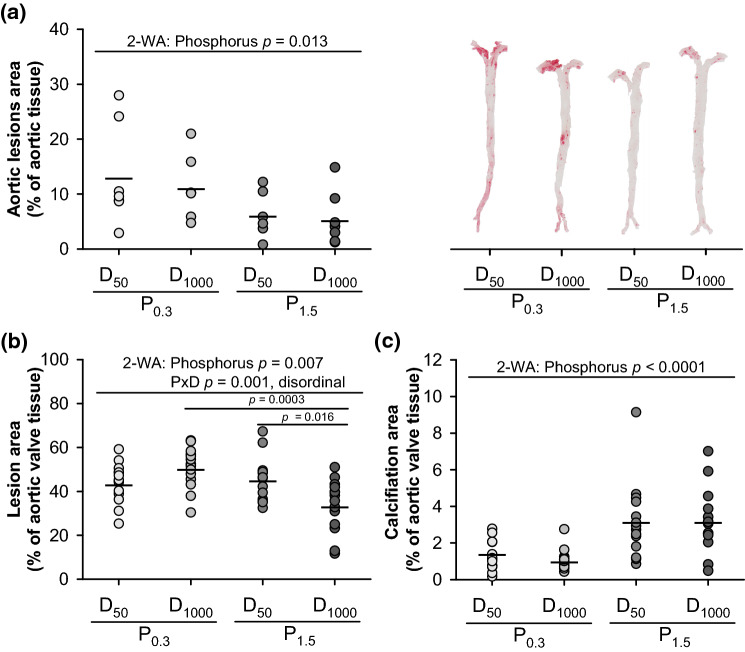
Histological analyses of the aorta and aortic valve of 26-week-old low-density lipoprotein receptor knockout (Ldlr^-/-^) mice. Mice were fed a Western diet with adequate (0.3%; P_0.3_) or high (1.5%; P_1.5_) phosphorus levels coupled with adequate (1000 IU; D_1000_) or low (50 IU; D_50_) vitamin D levels over a 17-week period. Shown are **(a)** the lesion areas of the entire aorta with representative images of *en face* prepared aortas that were stained by Oil red O, **(b)** the lesion area of the aortic valve tissue assessed by haematoxylin–eosin staining and **(c)** the calcification area of the aortic valve tissue assessed by von Kossa silver and Goldner I staining. Data are given as single (circle) and mean values (horizontal line) [n = 14 or n = 7 for **(a)**]. Data were analysed by two-factorial analysis of variance (2-WA) (significance level *p* < 0.05) with the classification factors phosphorus and vitamin D and their interaction.

To investigate the effect of a high-phosphorus diet on vascular lesion development in mice having an adequate and low vitamin D status, we quantified the lesion area of the entire aorta by the *en face* method and analysed the area and composition of vascular lesions in the aortic valve sections, including lipids, collagen, necrotic cores, macrophages and the calcified spots. Figure 1a demonstrates the strong effects of dietary phosphorus, but not of vitamin D, on the aortic lesion areas. Mice receiving the P_1.5_ diets had significantly smaller aortic lesions than mice fed the P_0.3_ diets (Fig. 1a). Data from the aortic valve section analysis demonstrate a significant interaction between dietary phosphorus and vitamin D on the lesion area. Aortic valves of mice fed the P_1.5_D_1000_ diet were characterised by smaller lesions than mice fed the P_0.3_D_1000_ or P_1.5_D_50_ diets (Fig. 1b). The results further indicate more calcification in the aortic valve tissue of mice fed the P_1.5_ diets than in those fed the P_0.3_ diets (Fig. 1c). However, dietary vitamin D did not influence aortic valve calcification. No effect of phosphorus and vitamin D was seen on the collagen area of the aortic valve (means ± SD in % aortic valve tissue: collagen: P_0.3_D_50_ 6.73 ± 4.31; P_0.3_D_1000_ 4.96 ± 2.63; P_1.5_D_50_ 7.07 ± 3.49; P_1.5_D_1000_ 8.35 ± 4.33; *p*-values P 0.0685, D 0.8036, PxD 0.134).

**Figure 2:**
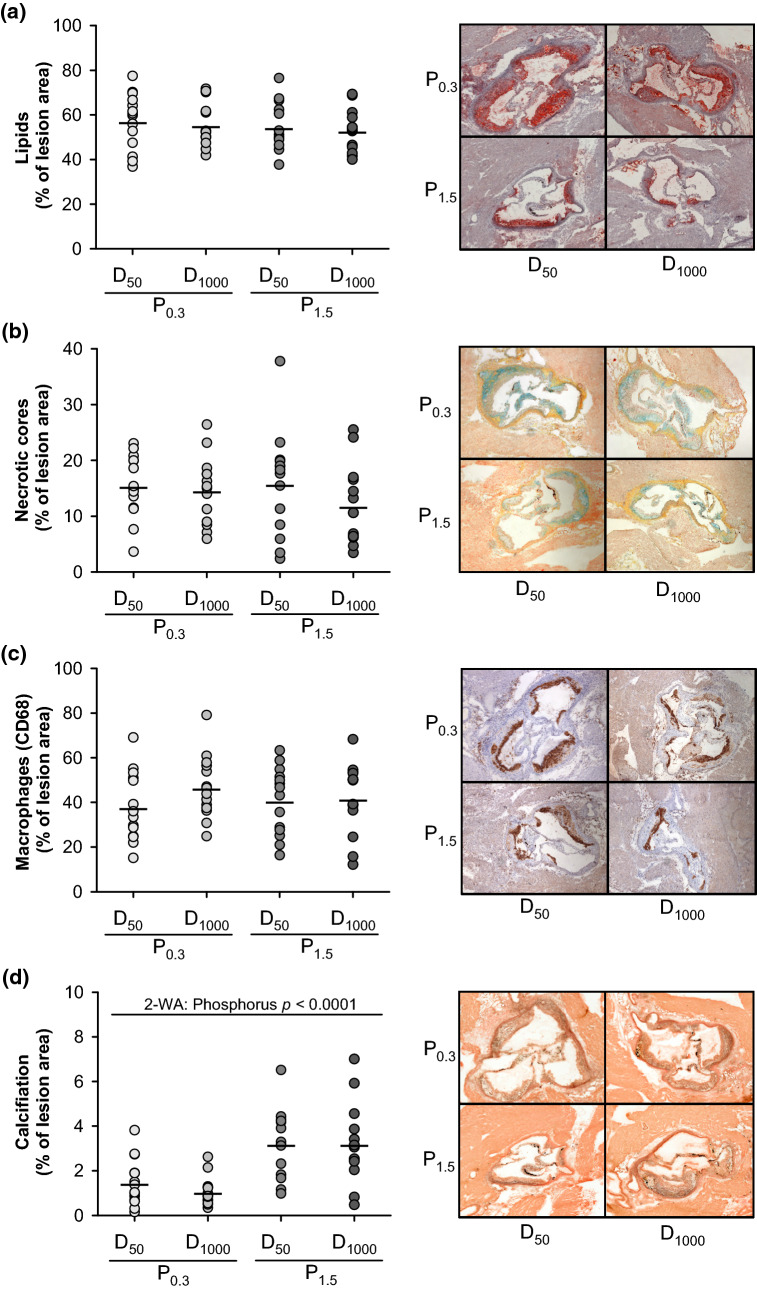
Histological analyses of atherosclerotic lesions in the aortic valve of 26-week-old low-density lipoprotein receptor knockout (Ldlr^-/-^) mice. Mice were fed a Western diet with adequate (0.3%; P_0.3_) or high (1.5%; P_1.5_) phosphorus levels coupled with adequate (1000 IU; D_1000_) or low (50 IU; D_50_) vitamin D levels over a 17-week period. Shown are **(a)** the percentage of lipid area in the aortic valve lesions and representative images of aortic valve sections that were stained by Oil Red O and haematoxylin, **(b)** the percentage of necrotic cores in the aortic valve lesion and representative images of aortic valve sections that were stained by Movat pentachrom staining, **(c)** the percentage of macrophages in the aortic valve lesions and representative images of aortic valve sections that were stained by immunohistochemical staining with CD68 antibodies and haematoxylin, and **(d)** the percentage of calcification in the aortic valve lesions and representative images of aortic valve sections that were stained by von Kossa silver and Goldner I. Data are given as single (CIRCLE) and mean values (HORIZONTAL LINE) [n = 14 or n = 7 for **(a)**]. Data were analysed by two-factorial analysis of variance (2-WA) (significance level *p* < 0.05) with the classification factors phosphorus and vitamin D and their interaction.

To ascertain whether dietary phosphorus and vitamin D can alter the lesion composition, we analysed the lipids, the necrotic cores, macrophages (CD68 positive cells) as well as the calcification in the lesions of the aortic valves. Analyses did not reveal any differences in the percentage area of lipid deposits, necrotic cores and macrophages per aortic valve lesion between the groups of mice (Fig. 2a–c). However, mice that received the P_1.5_ diets showed higher percentages of calcifications in their aortic valve lesions than mice fed the P_0.3_ diets (Fig. 2d).

### Cardiomyocyte morphology and regulators of heart growth

**Figure 3:**
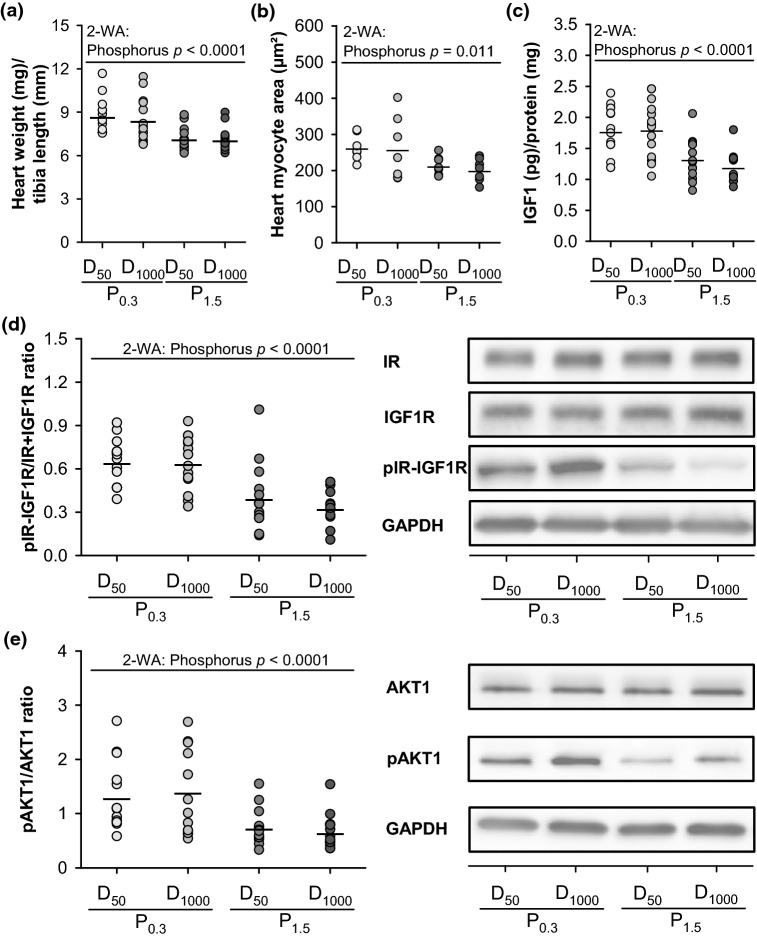
Heart weight (normalised to tibia length), cardiac myocyte size and regulators of heart growth of 26-week-old low-density lipoprotein receptor knockout (Ldlr^-/-^) mice. Mice were fed a Western diet with adequate (0.3%; P_0.3_) or high (1.5%; P_1.5_) phosphorus concentrations coupled with adequate (1000 IU; D_1000_) or low (50 IU; D_50_) concentrations of vitamin D over a 17-week period. Shown are **(a)** the heart weight, normalised to tibia length, **(b)** the cardiac myocyte area, **(c)** the insulin growth factor 1 (IGF1) concentration in the heart tissue, **(d)** the expression of phosphorylated insulin receptor-insulin growth factor 1 receptor dimer (pIR-IGF1R) in relation to IR and IGF1R expression with representative pictures of the western blot analysis, and **(e)** the expression of phosphorylated protein kinase B (pAKT1) in relation to AKT1 expression and representative images of the western blot analysis. Data are given as single (circle) and mean values (horizontal line) [n = 14 or n = 7 for **(b)**]. Data were analysed by two-factorial analysis of variance (2-WA) (significance level *p* < 0.05) with the classification factors phosphorus and vitamin D as well as their interaction.

The data show that dietary phosphorus, but not vitamin D, affected the heart weights of the mice. Irrespective of the vitamin D content in the diet, mice fed the P_1.5_ diets had a lower heart weight (normalised to tibia length) than mice fed the P_0.3_ diets (Fig. 3a). To elucidate whether the lower heart weights of the P_1.5_ groups were associated with a reduced cardiac myocyte size, we assessed the areas of cardiac myocytes. As depicted in Fig. 3b, cardiac myocytes of mice fed the P_1.5_ diet were smaller than those of mice fed the P_0.3_ diet. Dietary vitamin D did not influence the sizes of cardiac myocytes.

Because the IGF1-PI3-AKT signalling pathway is considered to be important for the proliferation and development of cardiac muscle cells, we analysed insulin-like growth factor 1 (IGF1) and RAC-alpha serine/threonine-protein kinase (AKT1). Here, we show that dietary phosphorus has a crucial impact on IGF1 expression in the heart; on the phosphorylation status of the insulin receptor (IR), the IGF1-receptor (IGF1R) and the IR-IGF1R dimer; and on AKT1 phosphorylation (Fig. 3c–e, Supplementary Fig. 1a). Dietary vitamin D had no effect on these parameters. Mice that received the P_1.5_ diets had a lower expression of IGF1 (Fig. 3c, Supplementary Fig. 1a) and lower levels of activated IR-IGF1R as determined by their phosphorylation state than mice fed the P_0.3_ diets (Fig. 3d, Supplementary Fig. 1a). In accordance with these findings, the levels of active AKT1 kinase, which is a downstream effector of IGF1, were lower in the P_1.5_ group than in the P_0.3_ group (Fig. 3e, Supplementary Fig. 1b). The expression of total AKT1, IGF1R and IR remained unaffected by the dietary treatments (Table 2, Supplementary Fig. 1a,b).

**Table 2:** Relative protein expression in the heart tissue of 26-week-old low-density lipoprotein receptor knockout (Ldlr^-/-^) mice.

Parameter	P_0.3_D_50_	P_0.3_D_1000_	P_1.5_D_50_	P_1.5_D_1000_	2-WA *p*-values		
					P	D	P × D
**Relative protein expression (reference protein: GAPDH)**							
IR	0.92 ± 0.17	1.00 ± 0.24	1.06 ± 0.22	1.11 ± 0.27	0.216	0.410	0.990
IGF1R	0.99 ± 0.25	1.01 ± 0.24	1.01 ± 0.39	1.08 ± 0.33	0.975	0.465	0.687
AKT1	1.00 ± 0.13	0.95 ± 0.20	1.05 ± 0.12	1.06 ± 0.15	0.322	0.625	0.596

Mice were fed a Western diet with adequate (0.3%; P_0.3_) or high (1.5%; P_1.5_) phosphorus levels coupled with adequate (1000 IU; D_1000_) or low (50 IU; D_50_) vitamin D levels over a 17-week period. The expression of the insulin receptor (IR), the insulin-like growth factor 1 receptor (IGF1R) and the RAC-alpha serine/threonine-protein kinase (AKT1) are shown.

Data are provided as mean ± standard deviation [n = 14]. Data were analysed via two-factorial analysis of variance (2-WA) (significance level *p* < 0.05) with the classification factors phosphorus and vitamin D as well as their interaction.

### Parameters of glucose metabolism

**Figure 4:**
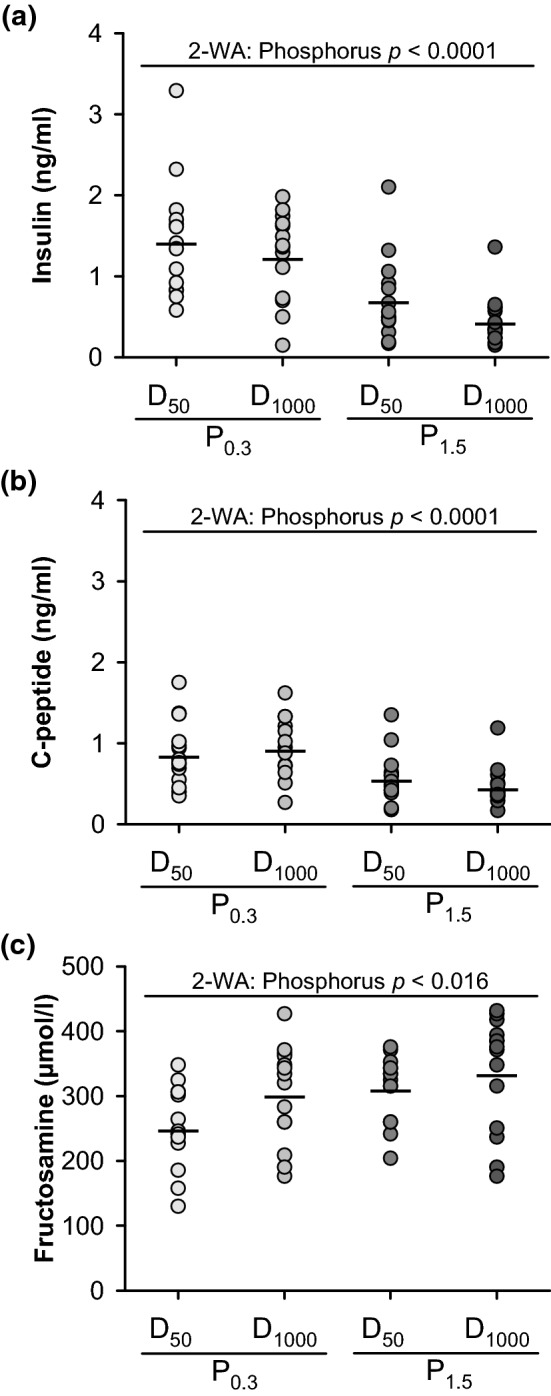
Serum concentrations of **(a)** insulin, **(b)** C-peptide and **(c)** fructosamine in 26-week-old low-density lipoprotein receptor knockout (Ldlr^-/-^) mice. Mice were fed a Western diet with adequate (0.3%; P_0.3_) or high (1.5%; P_1.5_) phosphorus concentrations coupled with adequate (1000 IU; D_1000_) or low (50 IU; D_50_) concentrations of vitamin D over a 17-week period. Data are given as single (circle) and mean values (horizontal line) [n = 14]. Data were analysed by two-factorial analysis of variance (2-WA) (significance level *p* < 0.05) with the classification factors phosphorus and vitamin D as well as their interaction.

In addition to the IGF1-PI3-AKT signalling pathway, insulin is another factor that may have influenced heart weight. Here, we found significant effects of dietary phosphorus on serum insulin concentrations (Fig. 4a). The P_1.5_ groups had significantly lower fasting concentrations of insulin and C-peptide, an indicator of insulin production, than the concentrations of the P_0.3_ groups (Fig. 4a,b). Dietary vitamin D displayed no effect on either parameter. To elucidate whether low insulin levels contributed to a higher glycation rate, we analysed serum concentrations of fructosamine. Analysis revealed that both groups of P_1.5_ had higher serum concentrations of fructosamine, a biomarker of hyperglycaemia, than those of the P_0.3_ groups (Fig. 4c). Fructosamine concentrations were not affected by vitamin D.

### Lipid profile

**Figure 5:**
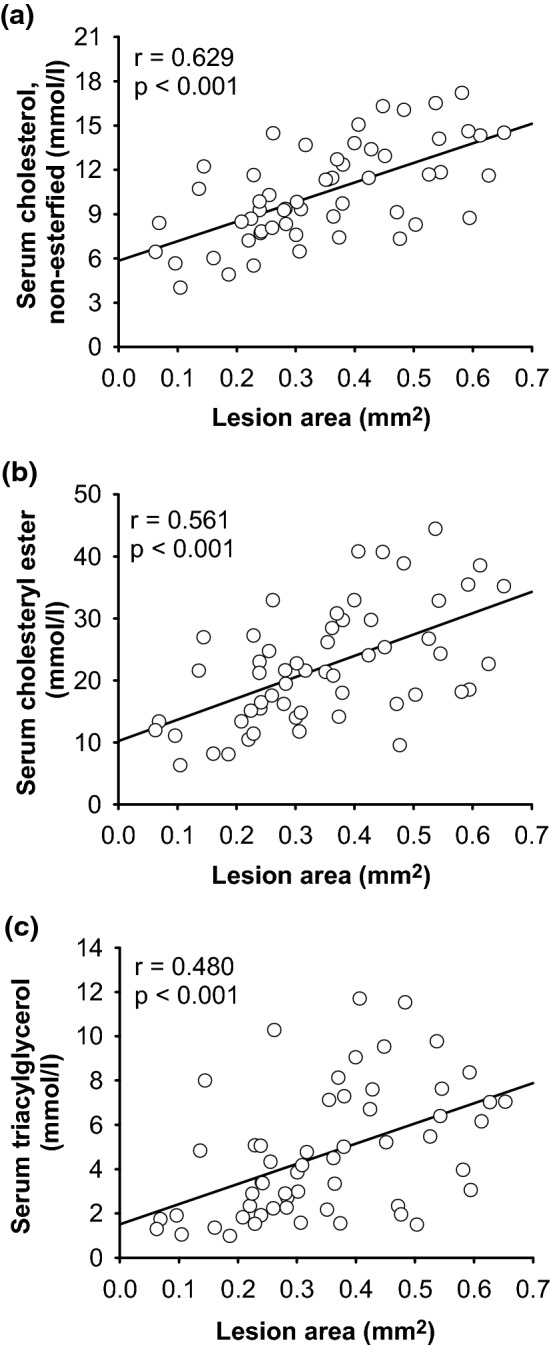
Correlation between serum **(a)** non-esterified cholesterol, **(b)** cholesteryl esters and **(c)** triacylglycerols and the total lesion area in the aortic valve of 26-week-old low-density lipoprotein receptor knockout (Ldlr^-/-^) mice. Mice were fed a Western diet with adequate (0.3%; P_0.3_) or high (1.5%; P_1.5_) phosphorus concentrations coupled with adequate (1000 IU; D_1000_) or low (50 IU; D_50_) concentrations of vitamin D over a 17-week period. Data from all mice were included in the correlation analysis [n = 56]. r = Pearson’s correlation coefficient (significance level *p* < 0.05).

Because serum lipids are important factors that can influence the development of aortic lesions, we analysed the serum concentrations of triacylglycerols (TAG), non-esterified cholesterol and cholesteryl esters. Analysis revealed that the serum concentrations of TAG and non-esterified cholesterol were influenced by dietary phosphorus and vitamin D. Mice that were fed the P_1.5_ diets had significantly lower serum concentrations of TAG, non-esterified cholesterol and cholesteryl esters than the concentrations of mice fed the P_0.3_ diets (Table 3). We were further able to show that mice that received the D_1000_ diets had lower serum concentrations of TAG and non-esterified cholesterol than those of mice fed the D_50_ diets, whereas the concentrations of cholesteryl esters remained unaffected (Table 3). Analyses revealed a positive correlation between the lesion area and the serum concentrations of cholesterol, cholesteryl esters and TAG (Pearson’s correlation coefficient for non-esterified cholesterol r = 0.629, cholesteryl esters r = 0.561, and TAG r = 0.480, with *p* < 0.001 for all) (Fig. 5). The strongest correlation was seen for the lesion area in the aortic valve and circulating non-esterified cholesterol. These correlations were also seen for the total lesion area in the entire aorta and the serum lipids (Pearson’s correlation coefficient for non-esterified cholesterol r = 0.668, *p* < 0.001; cholesteryl esters r = 0.631, *p* = 0.001; TAG r = 0.432, *p* = 0.028).

**Table 3:** Serum, liver, and faecal concentrations of lipids in 26-week-old low-density lipoprotein receptor knockout (Ldlr^-/-^) mice.

Parameter	P_0.3_D_50_	P_0.3_D_1000_	P_1.5_D_50_	P_1.5_D_1000_	2-WA *p*-values		
					P	D	P × D
**Serum in mmol/l**^1^							
Triacylglycerol	7.50 ± 2.01	5.84 ± 2.92	3.09 ± 1.56	2.25 ± 1.25	< .0001	0.026	0.455
Non-esterified cholesterol	13.0 ± 2.4	12.3 ± 2.8	9.1 ± 1.8	7.4 ± 2.0	< .0001	0.048	0.457
Cholesteryl ester	29.8 ± 7.7	26.7 ± 8.2	18.0 ± 6.5	14.2 ± 5.4	< .0001	0.073	0.843
**Liver in µmol/g liver**^1^							
Triacylglycerol	34.0 ± 32.7	60.2 ± 46.9*	28.8 ± 20.1	19.5 ± 9.6	0.052	0.391	0.033
Total cholesterol	36.1 ± 21.0	37.3 ± 13.7	25.0 ± 5.1	22.5 ± 7.5	< .0001	0.816	0.293
**Faeces in μmol/g faeces**^1^							
Non-esterified cholesterol	16.2 ± 8.4	18.9 ± 13.8	26.1 ± 10.4	29.8 ± 8.4	< .0001	0.361	0.712
Fatty acids	33.9 ± 20.0	41.3 ± 22.8	97.4 ± 71.3	98.0 ± 39.7	< .0001	0.663	0.697
Bile acids	164 ± 75	171 ± 108	85 ± 67	79 ± 36	0.000	0.986	0.739

Mice were fed a Western diet with adequate (0.3%; P_0.3_) or high (1.5%; P_1.5_) phosphorus concentrations coupled with adequate (1000 IU; D_1000_) or low (50 IU; D_50_) vitamin D concentrations over a 17-week period.

^1^Liver cholesterol and triacylglycerol was determined enzymatically, serum and faeces (except bile acids) parameters via high-performance thin-layer chromatography. Data are provided as mean ± standard deviation [n = 14]. Data were analysed via two-factorial analysis of variance (2-WA) (significance level *p* < 0.05) with the classification factors phosphorus and vitamin D as well as their interaction. *differs from P_1.5_D_1000._

To elucidate whether the changes in serum lipid concentrations were accompanied by changes in tissue concentrations of lipids, we quantified the TAG and total cholesterol in the liver. Analyses revealed a significant interaction between dietary phosphorus and vitamin D on liver TAG. As shown in Table 3, the highest TAG concentration in the liver was found in the P_0.3_D_1000_ group, and the lowest concentration was seen in livers from the P_1.5_D_1000_ group (Table 3). Concentrations of liver cholesterol were also affected by dietary phosphorus but not by vitamin D. According to serum cholesterol, the concentration of cholesterol in the liver was lower in the P_1.5_ groups than in the P_0.3_ groups (Table 3). To investigate whether the changes in serum lipid concentrations were caused by alterations in faecal excretion of lipids, we quantified lipids and bile acids in faeces samples of mice. Here, we found marked effects of dietary phosphorus, but not vitamin D, on faecal lipid and bile acid excretion. Faecal excretion of non-esterified cholesterol and fatty acids was higher in the P_1.5_ groups than in the P_0.3_ groups (Table 3). In contrast, the excretion of bile acids was lower in the P_1.5_ group than in the P_0.3_ group (Table 3).

To elucidate whether the observed changes in cholesterol concentrations and bile acid excretion are associated with an altered mRNA abundance of hepatic transporters and regulators that are involved in cholesterol homeostasis, we analysed the mRNA expression of ATP binding cassette subfamily G members 5 and 8 (Abcg5, Abcg8) and subfamily B member 11 (Abcb11) as well as subfamily C member 2 (Abcc2), the nuclear receptor subfamily 1 group H member 3 (Nr1h3) and subfamily 0 group B (Nr0b2), which are important regulators of cholesterol, and the cytochrome P450/family 27/subfamily a/polypeptide 1 (Cyp27a1) and family 7/subfamily a/polypeptide 1 (Cyp7a1), which are involved in the conversion of cholesterol to bile acids. Additionally, we measured the mRNA abundance of the lipoprotein receptors scavenger receptor class B, member 1 (Scarb1), low density lipoprotein receptor-related protein 1 (Lrp1) and 3-hydroxy-3-methylglutaryl-Coenzyme A reductase (Hmgcr). The findings demonstrate that the mRNA abundance of Abcg5, Abcg8 and Nr1h3, Nr0b2 as well as Lrp1 and Scarb1 in the livers of mice that received the P_1.5_ diets was significantly lower than in the livers of mice fed the P_0.3_ diets (Table 4). The mRNA abundance of these genes remained unaffected by vitamin D. The relative gene expression of Abcb11, Abcc2, Cyp7a1, Cyp27a1 and Hmgcr3 in the liver was not altered by the dietary treatments (Table 4).

**Table 4:** Relative mRNA abundance of genes involved in cellular lipid uptake and cholesterol metabolism in 26-week-old low-density lipoprotein receptor knockout (Ldlr^-/-^) mice.

Parameter	P_0.3_D_50_	P_0.3_D_1000_	P_1.5_D_50_	P_1.5_D_1000_	2-WA *p*-values		
					P	D	P × D
**Relative mRNA expression**							
Abcc2	0.92 ± 0.29	1.00 ± 0.26	0.94 ± 0.29	0.96 ± 0.32	0.839	0.444	0.671
Abcg5	1.09 ± 0.24	1.00 ± 0.26	0.80 ± 0.28	0.85 ± 0.12	0.0003	0.725	0.215
Abcg8	1.10 ± 0.29	1.00 ± 0.23	0.73 ± 0.28	0.75 ± 0.14	< .0001	0.501	0.318
Acbc11	1.11 ± 0.43	1.00 ± 0.39	0.78 ± 0.17	0.95 ± 0.30	0.077	0.641	0.146
Cyp27a1	1.01 ± 0.15	1.00 ± 0.15	1.02 ± 0.30	1.03 ± 0.24	0.776	0.965	0.807
Cyp7a1	1.18 ± 1.04	1.00 ± 0.77	0.74 ± 0.50	0.86 ± 0.56	0.144	0.944	0.439
Hmgcr3	1.20 ± 0.43	1.00 ± 0.43	1.12 ± 0.53	1.48 ± 0.80	0.505	0.791	0.090
Lrp1	0.85 ± 0.35	1.00 ± 0.40	0.69 ± 0.20	0.66 ± 0.21	0.006	0.519	0.308
Nr0b2	0.86 ± 0.44	1.00 ± 0.28	0.69 ± 0.34	0.72 ± 0.25	0.041	0.153	0.470
Nr1h3	0.94 ± 0.12	1.00 ± 0.19	0.87 ± 0.27	0.89 ± 0.16	0.038	0.346	0.636
Scarb1	1.07 ± 0.47	1.00 ± 0.34	0.63 ± 0.23	0.73 ± 0.27	0.0003	0.537	0.473

Mice were fed a Western diet with adequate (0.3%; P_0.3_) or high (1.5%; P_1.5_) phosphorus concentrations coupled with adequate (1000 IU; D_1000_) or low (50 IU; D_50_) vitamin D concentrations over a 17-week period.

Data are provided as mean ± standard deviation [n = 14]. Data were analysed via two-factorial analysis of variance (2-WA) (significance level *p* < 0.05) with the classification factors phosphorus and vitamin D as well as their interaction. ATP-binding cassette, sub-family C (CFTR/MRP), member 2 (Abcc2), ATP-binding cassette, sub-family B (MDR/TAP), member 11 (Acbc11), cytochrome P450, family 27, subfamily a, polypeptide 1 (Cyp27a1), cytochrome P450, family 7, subfamily a, polypeptide 1 (Cyp7a1), 3-hydroxy-3-methylglutaryl-coenzyme A reductase (Hmgcr), hypoxanthine guanine phosphoribosyl transferase (Hprt), low density lipoprotein receptor-related protein 1 (Lrp1), nuclear receptor subfamily 0, group B, member 2 (Nr0b2), nuclear receptor subfamily 1 group H member 3 (Nr1h3), scavenger receptor class B, member 1 (Scarb1).

## Discussion

The current study aimed to investigate the impact of dietary phosphorus and vitamin D on vascular lesion development and heart morphology in a two-factorial study with Ldlr^-/-^ mice as an atherosclerosis model.

Here, we found that high-phosphorus diets had a stronger impact on vascular lesion development and cardiac morphology than an inadequate vitamin D supply, which was seen in reduced serum concentrations of 25(OH)D. Importantly, we observed only a few interactions between phosphorus and vitamin D. The main findings were that high-phosphorus diets reduced the aortic lesion areas, without a concomitant change in the plaque composition of lipids, necrotic cores and macrophages, increased the vascular calcification, and induced cardiac hypotrophy. These changes were independent of the vitamin D status, which was assessed by the analysis of serum 25(OH)D and 1,25(OH)_2_D. Assessing the atherosclerotic lesions in the cardiac valves, the group that received the high-phosphorus and vitamin D-adequate diet had the lowest lesion areas. The analysis of the aortic valve collagen and lesion composition showed no impact of phosphorus or vitamin D on lipid deposition, necrotic core area, and macrophages, indicating no phosphorus- or vitamin D-specific effects on these parameters, which are indicative of the stability and progression of atherosclerotic plaques.

Evidence that hyperphosphataemia promotes vascular calcification comes from clinical studies with patients suffering from CKD^9,30,31^ and from hyperphosphataemic FGF23-deficient mice^32^. The current data clearly show that vascular calcification also occurs in phosphorus-treated mice, although their fasting levels of phosphate were not increased. Thus, it can be assumed that dietary phosphorus has the potential to stimulate vascular calcification, even in those having an intact phosphorus regulating system. The data clearly show that the increased secretion of PTH and iFGF23, which stimulate the renal phosphate excretion, can keep the circulating fasting levels of phosphate within a normal range. However, in a previous human post-meal study, in which healthy subjects received a phosphate supplement or a placebo, the phosphate intervention was associated with higher postprandial phosphate levels than the placebo treatment^4^. This finding indicates that the post-meal levels of phosphate increase after phosphate intake but are normalised within a few hours postprandial by the phosphaturic hormones.

Unexpectedly, we found smaller valvular and aortic lesions as well as smaller hearts in mice fed the high-phosphorus diets. This contrasts with data that found higher serum phosphorus concentrations associated with a higher left ventricular mass in men^16^ and subclinical atherosclerosis in the general population^33^. To elucidate the possible mechanism that may explain the current findings, we analysed iFGF23 and iPTH, which are involved in the regulation of phosphorus metabolism and can modulate cardiovascular risk^34–36^. Here, we found that the levels of iFGF23 were 6.5-fold higher and the levels of iPTH were threefold higher in mice fed the high-phosphorus diets than in mice fed the adequate-phosphorus diets. As several studies found high FGF23 concentrations associated with myocardial hypertrophy^37–39^ and high serum concentrations of PTH related to atherogenesis and heart failure in the general population^40,41^, we would have expected more atherosclerotic lesions and a higher cardiac mass in the high-phosphorus group than in the adequate-phosphorus group. Interestingly, Shiota et al. (2011), who conducted a study with apolipoprotein E-deficient (ApoE^-/-^) mice, also found smaller atherosclerotic lesions in mice fed high-phosphorus diets^42^. Ldlr^-/-^ and ApoE^-/-^ mice, which are normally used as an atherosclerosis model, are characterised by extremely high levels of lipids in the serum. To elucidate whether phosphorus influenced lipid metabolism, which may explain the reduced vascular lesion areas, we analysed the serum lipids in these mice and found markedly reduced serum concentrations of TAG, non-esterified cholesterol and cholesteryl esters in mice fed the high-phosphorus diets. Thus, we speculate that the pronounced lipid-lowering effect of dietary phosphorus provides a possible explanation for the reduction in vascular lesions in the high-phosphorus groups. This assumption is supported by the finding of positive correlations between serum lipids and the lesion areas in the aortic valve and aorta, respectively.

To determine the mechanisms of the phosphorus-mediated effect on serum lipids, we measured lipids in faeces and found a higher excretion of fatty acids and cholesterol in mice fed the high-phosphorus diets than in those fed the adequate-phosphorus diets. Thus, we assume that the reduced serum and liver concentrations of lipids were, at least in part, caused by a higher intestinal loss of these lipids. It has already been described that precipitated calcium and phosphorus in the small intestine can form insoluble amorphous calcium phosphate complexes that can bind and inactivate luminal bile acids, thereby lowering the serum cholesterol level^43,44^. However, the current study shows a lower faecal excretion of bile acids in the high-phosphorus group than in the adequate-phosphorus group. This finding fits well with the observation that the high-phosphorus groups had a reduced hepatic mRNA abundance of Nr1h3, a nuclear receptor that functions as a cholesterol sensor and that stimulates the conversion of cholesterol into bile acids^45^. The high-phosphorus groups were also characterised by a higher mRNA expression of Nr0b2, which modulates the transcriptional activity of Nr1h3^46^. The lower mRNA abundance of Nr1h3 in the liver was further accompanied by a lower expression of the hepatic sterol transporters Abcg5/g8 in the liver, which mediate cholesterol excretion into the bile canaliculus^47^. As Nr1h3 is also an important stimulator of hepatic lipogenesis^48,49^, we assume that the reduced serum and liver concentrations of TAG in mice fed the high-phosphorus diets were caused by the low mRNA abundance of Nr1h3. Additionally, mice fed the high-phosphorus diets showed a lower mRNA expression of the lipoprotein receptors Scarb1 and Lrp1, which facilitate the cellular uptake of high-density lipoproteins, LDL and chylomicrons. These finding also fits well to the observed reductions of serum and liver lipids in mice fed the high-phosphorus diets.

This is in accordance with data from a recent study that observed a downregulation of hepatic genes involved in lipogenesis in rats fed a high-phosphorus diet^50^. As insulin can stimulate lipogenesis, we assume that the reduced lipid concentrations in the liver of mice fed the high-phosphorus diets could partly be due to the reduced insulin serum concentrations that were observed in these mice. Interestingly, circulating lipid levels were also found to be influenced by dietary vitamin D, in such a way that mice that received the vitamin D-adequate diets had lower serum concentrations of TAG and non-esterified cholesterols than those of mice fed the low-vitamin D diets. This finding is in accordance with recent studies that reported an association between vitamin D inadequacy and hyperlipidaemia^51^, although a mechanism that may explain these vitamin D effects could not be found so far. The lowest serum lipid concentrations were found in the high-phosphorus, vitamin D-adequate group. This group was also characterised by the lowest atherosclerotic lesion areas in the aortic valve. In addition to the phosphorus effect, there was an interaction between phosphorus and vitamin D regarding the TAG concentration in the liver. The group receiving an adequate amount of vitamin D and phosphorus had the highest levels of cholesterol and TAG in the liver.

An important finding was the very high serum concentration of 1,25(OH)_2_D in the P_1.5_D_1000_ group in comparison to the other groups. Many studies report 1,25(OH)_2_D serum concentrations of mice receiving adequate vitamin D diets to be 200 ± 20 pmol/l^52–54^, which indicates that the P_1.5_D_1000_ group in our study had comparatively high levels of 1,25(OH)_2_D. Elevated calcitriol serum concentrations have also been observed in young adults who consumed a diet high in phosphorus and low in calcium^55^. The reduced serum calcium concentration in combination with available dietary vitamin D could have contributed to an increased synthesis of calcitriol in the liver to increase calcium absorption in the intestine^55^.

To determine the possible mechanisms that may explain the smaller hearts in the high-phosphorus groups, we measured regulators of cardiac myocyte growth. One of these growth regulators is IGF1. Myocardial cells have been shown to produce IGF1, which acts locally in an autocrine manner ^56,57^. Here, we show that IGF1 expression in the hearts of mice that received the high-phosphorus diets was lower than that in the hearts of mice that were fed the adequate-phosphorus diets. IGF1 regulates cell growth via IGF1R, which is autophosphorylated to pIGF1R after IGF1 binding. One of the downstream targets of the IGF/IGFR pathway is AKT1, which in its phosphorylated form (pAKT1, Ser473) can stimulate heart growth. In addition to the lower cardiac expression of IGF1 observed in the high-phosphorus groups, the results further show that these mice also had lower cardiac expression of pIR-IGF1 and pAKT, which could have contributed to the lower heart weight and the smaller cardiac myocytes. Another molecule that could have contributed to cardiac hypotrophy is insulin. Similar to IGF1, insulin that is bound to IR can stimulate the phosphorylation of AKT1 to pAKT1. IR and IGFR can heterodimerize to form a hybrid receptor^58^. Here, we found that the serum concentrations of insulin and its cleavage product C-peptide were markedly lower in the high-phosphorus groups (-50%) than in the adequate-phosphorus groups. The reduced levels of C-peptide indicate that the reduced insulin levels were caused by a decreased synthesis of insulin. As insulin is the master regulator of serum glucose, we analysed the glycaemic biomarker fructosamine and found higher fructosamine concentrations in the serum of the high-phosphorus groups than in the adequate-phosphorus groups, indicating that the amount of secreted insulin was inefficient to normalize glucose levels in these mice. In vitro studies have shown that phosphorus can cause oxidative stress in the mitochondria of pancreatic beta cells, which in turn leads to cytotoxicity and deteriorated insulin production^59^. This might explain the observed effect of the high-phosphorus diets on serum concentrations of insulin.

To date, only a few human studies have been conducted to evaluate the effects of phosphorus on lipid and glucose metabolism in healthy subjects. In a small study including eight healthy men, phosphorus supplementation increased postprandial levels of apolipoprotein B48 and decreased those of apolipoprotein B100^60^. In accordance with our findings, data from a cross-sectional study showed that high plasma levels of phosphate were associated with low levels of circulating TAG and insulin^61^. However, in contrast to the current study, they found high levels of circulating phosphate associated with high plasma concentrations of cholesterol. To conclude, the present study demonstrates that a high intake of phosphorus may stimulate vascular calcification but reduce the formation of vascular lesions. Dietary phosphorus further induced cardiac hypotrophy, which was probably induced by low levels of the IGF1 and insulin signalling pathway. The study further showed that dietary phosphorus increased the faecal excretion of cholesterol. High-phosphorus and vitamin D-adequate diets can contribute to lowering the circulating concentrations of TAG and cholesterol.

## Methods

### Mice and diets

The mouse study described in this manuscript followed the established guidelines of the American Physiological Society and was approved by the local government (Landesverwaltungsamt Sachsen-Anhalt, Germany, approval number: 42502-2-1403 MLU). All mice were housed pairwise in Macrolon cages in a room with controlled temperature (22 ± 1 °C), humidity (50–60%), and lighting (6 am–6 pm) and had free access to food and water.

The study was conducted in a two-factorial design with the factors dietary phosphorus and vitamin D. Fifty-six 9-week-old male Ldlr^-/-^ mice (B6.129S7-Ldlr^tm1Her^/J; The Jackson Laboratory, Bar Harbor, ME, USA) were randomly assigned to four experimental groups (n = 14). Mice were fed diets with adequate (0.3%; P_0.3_) or high (1.5%; P_1.5_) phosphorus concentrations combined with either adequate (1000 IU/kg diet; D_1000_) or low (50 IU/kg diet; D_50_) contents of vitamin D_3_, for 17 weeks. To stimulate vascular lesion development, mice received a Western diet rich in sugar, fat and cholesterol (in g/kg diet: starch 241.5, casein 200, sucrose 200, lard 210, vitamin and mineral mixture 90, cellulose 50, soybean oil 5, DL-methionine 2, cholesterol 1.5). Vitamins and minerals, with the exception of vitamin D_3_, were added to the diets according to the recommendations of the American Institute of Nutrition (AIN)^62^. Phosphorus was added as sodium dihydrogen phosphate monohydrate (NaH_2_PO_4_∙H_2_O). Adequate-phosphorus diets contained sodium carbonate decahydrate (Na_2_CO_3_∙10 H_2_O) to achieve equal amounts of sodium in all diets.

### Sample collection

At the age of 26 weeks, mice were food deprived for 4 h, anaesthetized with diethyl ether, and sacrificed. Blood was collected into microtubes (Sarstedt, Nümbrecht, Germany) to obtain serum. The hearts with the ascending part of the aorta were prepared, rinsed with cold 0.9% NaCl solution and weighed. After separation of the apex, the heart with the adhering aortic root and the apex were snap-frozen in liquid nitrogen. The aorta (from the aorta brachiocephalica to the iliac bifurcation) of seven mice per group was excised and carefully cleaned of adhering fat as well as connective tissue and fixed in 4% paraformaldehyde for *en face* analysis. Livers were harvested and snap-frozen in liquid nitrogen. Faeces were collected from the rectum. Serum, tissues, and faeces were stored at − 80 °C until analyses.

### Analysis of 25(OH)D and 1,25(OH)_2_D in serum

The serum concentration of 25(OH)D was quantified by high-pressure liquid chromatography (Agilent 1260, Agilent Technologies, Waldbronn, Germany) coupled to a QTRAP5500-mass spectrometry system (AB Sciex, Darmstadt, Germany) with a commercially available kit (MassChrom 25-OH-vitamin D_3_/D_2_ in serum/plasma—LC–MS/MS reagent kit, Chromsystems Instruments & Chemicals GmbH, Gräfelfing, Germany) as described in detail in Baur et al. 2019^63^. The coefficient of variation was 6.9% at a concentration of 40.8 nmol/l, and the limit of quantification (LOQ) was 7.5 nmol/l. The serum concentration of 1,25(OH)_2_D was measured using IDS 1,25-dihydroxy vitamin D EIA following the manufacturer’s protocol (Immunodiagnostic Systems GmbH, Frankfurt, Germany). To reduce the amount of serum required, the manufacturer’s instructions were slightly modified, a 1:3 dilution of the serum was made, and 50 μl was used instead of 100 μl of the samples and controls for the immune extraction.

### Analysis of minerals, hormones and fructosamine in serum

The serum concentrations of ionic calcium, inorganic phosphate and fructosamine were measured spectrophotometrically according to the manufacturer’s protocols (Fluitest CA, Fluitest PHOS, Fluitest FRUC, Analyticon Biotechnologies AG, Lichtenfels, Germany). Hormones were quantified with the use of ELISA kits: iPTH (#60-2305, Immunotopics, San Clemente, CA, USA), iFGF23 (#60-6800; Immunotopics), insulin (Mouse Insulin ELISA #10-1247-01, Mercodia Uppsala, Sweden), C-peptide (Mouse C-Peptide ELISA Kit #90050, Crystal Chem, Inc., IL, USA) and IGF1 (Quantikine ELISA Mouse/Rat IGF-I Immunoassay #MG100, R&D Systems, Inc., MN, USA) according to the manufacturers’ specifications.

### Quantification and characterization of vascular lesions

To quantify total aortic lesions, the aortas were stained by Oil Red O according to the method of Andrés-Manzano et al. 2015^64^. Stained aortas were cut lengthwise and opened. Aortic lesion areas were quantified (Axiovert 200 microscope and AxioCamMRc using Axiovision Rel. 4.8.2 software, Carl Zeiss, Jena, Germany).

From hearts with the adjacent aortic arch, serial 10 µm thick slices (CM 1850 UV microtome, Leica, Nussloch, Germany) of the aortic valve were prepared. The following staining methods were used for the determination of plaque size and composition of the aortic valve tissue and the lesions localised in the aortic valves: Oil Red O staining was used to visualise vascular lipids (counterstaining: haematoxylin (Dr. Hollborn & Söhne GmbH & Co.KG., Leipzig, Germany), and von Kossa silver method, counterstained with Goldner I (Carl Roth, Karlsruhe, Germany) was used to visualise vascular calcification. Infiltration of macrophages in the aortic valve was assessed by immunohistochemistry^65^ using the primary antibody against CD68 (CD68 + rat anti mouse, monoclonal (AbD Serotec, Oxford, UK) and the horseradish peroxidase-labelled secondary antibody (goat anti-rat IgG (AbD Serotec)). For further characterization of the collagen and necrotic cores in the aortic valves, Movat-pentachrom staining kit (Morphisto GmbH, Frankfurt am Main, Germany) was used according to the manufacturers protocol. The atherosclerotic lesions were quantified histomorphometrically (Axiovert 200 microscope, AxioCamMRc, Axiovision Rel. 4.8.2 software). The aortic valve tissue was calculated from the cross-section area of the aortic valve minus the lumen and was used to assess the percentage area of lesions and calcification. The total lesion area in the aortic valve was used for the analysis of the lesion composition.

### Morphology of the heart

The blind sample analysis of the cardiomyocyte sizes from the cardiac apex was carried out as described recently^66^. In brief, after rinsing the cardiac tissue with phosphate-buffered saline (PBS) and dehydration, samples were embedded in PEG 1500 (Merck-Schuchhardt, Hohenbrunn, Germany). Thereafter, sections of 2 µm thickness were prepared (Microm HM 335 E, Thermo Fisher Scientific, Waltham, MA, USA) and stained with haematoxylin (Thermo Fisher Scientific) prior to analysis of myofibril area (Axiovert 200 microscope, AxioCamMRc, Axiovision Rel. 4.8.2 software).

### Western blot analysis of the heart

The preparation of the heart samples and the western blot analysis were performed as described elsewhere in detail^66^. Glyceraldehyde-3-phosphate dehydrogenase (GAPDH), which remained unaffected by the dietary treatments, was used for normalization of protein expression data. The mean GAPDH signal intensity of each gel was used to normalise for differences in the signal intensity between the individual western blot gels.

The following antibodies were used: anti-insulin receptor (#3025, Cell Signaling Technology (CST), Danvers, MA, USA), anti-insulin-like growth factor 1 receptor (#9750, CST), phosphoIR/IGF1R (#3024, CST), anti-AKT1 (#4691, CST), anti-phosphoAKT1 (#4060 and #9018, CST), and anti-GAPDH (#5174, CST). Primary antibodies were detected using HRP-conjugated secondary antibodies (anti-rabbit IgG, (#7074, CST) or anti-mouse (#7076, CST)) using ECL Prime Western blotting detection reagent (GE Healthcare, Munich, Germany).

Detection and visualization of the primary antibodies were performed as described elsewhere^66^. The density of each band which was corrected for protein loading using the reference protein was measured with a computer-assisted imaging analysis system (Gene Tools, Syngene, Cambridge, UK). Data on the protein expression were shown as relative protein abundance (relative to the reference protein). Samples from all groups were applied alternately on each blot and the reference protein was incubated on the same blot as standard. Blots signals were detected with Syngene G-Box and Genesys V.1.4.00 and analysed using the GeneTools from Syngene 4.03.02.0 software.

### Quantification of faecal bile acids

Faecal bile acids were quantified by use of a photometric test system for the in vitro determination of bile acids in faeces (IDK Bile Acids Test, Immundiagnostik AK, Bensheim, Germany). Prior to analysis, faeces were freeze-dried and then homogenised in a tissue lyser.

### Analysis of lipids in serum, liver, and faeces

Lipid extracts from liver were prepared as described elsewhere^67^, and the concentrations of TAG and cholesterol in lipid extracts were quantified using enzymatic reagent kits according to the manufacturer’s protocol (DiaSys Diagnostic Systems GmbH, Holzheim, Germany). Lipids in serum and faeces were quantified by high-performance thin-layer chromatography (HPTLC). Prior to that, serum and freeze-dried faeces were extracted with chloroform:methanol (2:1, v:v). Mixtures of cholesterol (Sigma-Aldrich Chemie GmbH, Steinheim, Germany), cholesteryl oleate (Sigma-Aldrich), soy oil (Kunella Feinkost GmbH, Cottbus, Germany) and cis-10-heptadecenoic acid (Santa Cruz Biotechnology Inc., Dallas, TX, USA) dissolved in chloroform:methanol (2:1, v:v) were used as standards. HPTLC plates (HPTLC Silica Gel 60, Merck KGaA, Darmstadt, Germany) were loaded using 1-µl capillary pipettes, and n-hexane:diethyl ether:acetic acid (40:10:1.5, v:v:v) was used as the solvent carrier. The plates were air dried and immersed in 10% (w/v) copper sulfate (dissolved in 8% (v/v) phosphoric acid) for 3 s and then developed for 11 min at 160 ± 3 °C. The plates were scanned with a colour scanner, and the images were assessed for grey values of baseline corrected peaks using Multi Gauge V3.1 Software (Fujifilm Life Science, Tokyo, Japan). The lipids were quantified by nonlinear regression using a 2-parameter equation for the exponential increase to the maximum. The LOQs and the coefficients of variation (in parentheses) determined for applied amounts of 0.6 µg of individual lipids on the plate were 3.90 ng (4.5%) for cholesterol, 26.1 ng (3.2%) for cholesteryl oleate, 15.1 ng (3.7%) for TAG and 29.6 ng (3.7%) for cis-10-heptadecenoic acid.

### RNA isolation and real-time RT-PCR

Total RNA was extracted from the liver using peqGOLD TriFast reagent (VWR International LLC, Radnor, PA, USA) according to the manufacturer’s protocol. Total RNA concentrations and purities were estimated by measuring the optical density at 260 and 280 nm, respectively. A total of 1.2 µg of total RNA was used for cDNA synthesis with M-MLV reverse transcriptase (Promega, Madison, WI, USA). Real-time RT-PCR analyses were performed as described elsewhere in detail^68^. For the determination of mRNA concentration, a threshold cycle (Ct) and amplification efficiency were obtained from each amplification curve using Rotor-Gene software version 4.6 (Corbett Research, Mortlake, Australia). The relative mRNA concentration was calculated according to Pfaffl 2001^69^. Several reference genes were analysed, and their stable expression levels in all groups were evaluated by calculating Ct values. The most stable reference gene was used for normalisation. This reference gene was hypoxanthine guanine phosphoribosyl transferase (Hprt). Primer pairs for Hprt (NM_013556), Cyp27a1 (NM_024264) and Nr1h3 (NM_001177730) were purchased from Sigma-Aldrich (KiCqStart SYBR Green Primers). The sequences of the other primers used for PCR (Eurofins Genomics, Ebersberg, Germany) were as follows: Abcg5 (NM_031884) forward 5′-TGGATCCAACACCTCTATGCTAAA-'3, reverse 5′-GGCAGGTTTTCTCGATGAACTG-'3, Abcg8 (NM_026180) forward 5′-TGCCCACCTTCCACATGTC-'3, reverse 5′-ATGAAGCCGGCAGTAAGGTAGA-'3, Abcb11 (NM_001363492.1) forward 5′-ATTAACCTTGGTGATTCTCG-'3, reverse 5′-ATACCTTTCCACCTCCTTATTC-'3, Abcc2 (XM_006526625.4) forward 5′-CGTATATAAGAAGGCACTAACC-'3, reverse 5′-R: CAATCTGTAAAACACTGGACC-'3, Cyp7a1 (NM_007824.2) forward 5′-TCAAGCAAACACCATTCCTGCAAC-'3, reverse 5′-R: TTCAGGATCCAAGTGCATTAACTG, Hmgcr (NM_008255.2) forward 5′-TGGAGATCATGTGCTGCTTC-'3, reverse 5′-GCGACTATGAGCGTGAACAA-'3, Lrp1 (NM_008512.2) forward 5′-CCTACCTAGACTACATCGAG-'3, reverse 5′-GCGTAGAGATAGTTCTCAAAC-'3, Nr0b2 (NM_011850.3) forward 5′-CGTCCTCTTCAACCCAG-'3, reverse 5′-GAGATCTACCAGAAGGGTG-'3, Scarb1 (NM_016741.1) forward 5′-GTCCGCATAGACCCGAGCAG-'3, reverse 5′-CCAGCGCCAAGGTCATCATC-'3.

### Statistics

The statistical analysis was carried out with the software SAS Studio (SAS OnDemand for Academics used last 09.01.2020). All parameters were tested for normal distribution using the Kolmogorov–Smirnov test and the quantile–quantile diagram and, if necessary, log-transformed. Homogeneity of variance was tested with Levene’s test. A two-factorial analysis of variance was used to compare the effects of dietary phosphorus (0.3% vs. 1.5%), vitamin D (50 IU/kg vs. 1000 IU/kg) and their interactions. The significance level was set at *p* < 0.05. In the case of a significant interaction between the two dietary factors (tested for ordinal, semiordinal or disordinal interactions), a post hoc Tukey test or Bonferroni test was used. Pearson’s correlation coefficient (r) was used to evaluate the correlation between two variables.

## Supplementary Information

Supplementary Figure S1.

## Data Availability

The datasets generated during and/or analysed during the current study are available from the corresponding author on reasonable request.
