# Supplementary material for: High-phosphorus diets reduce aortic lesions and cardiomyocyte size and modify lipid metabolism in Ldl receptor knockout mice
Source: Sci Rep. 2020 Nov 27;10:20748. doi: 10.1038/s41598-020-77509-w (PMC7695849; doi:10.1038/s41598-020-77509-w)
Supplement: Supplementary file 1 — Supplementary Figure S1. [file 41598_2020_77509_MOESM1_ESM.pdf]

Supplementary Information

High-phosphorus diets reduce aortic lesions and cardiomyocyte size and modify lipid metabolism in Ldl receptor knockout mice

Sarah M. Grundmann[1,2], Alexandra Schutkowski[1], Christian Berger[1], Anja C. Baur[1,2], Bettina König[1,2], Gabriele I. Stangl[1,2*]

[1] Institute of Agricultural and Nutritional Sciences, Martin Luther University Halle-Wittenberg, Halle (Saale), Germany

[2] Competence Cluster for Nutrition and Cardiovascular Health (nutriCARD), Halle-Jena-Leipzig, Germany

[*] Corresponding author:

Gabriele I. Stangl

Gabriele.Stangl@landw.uni-halle.de

Institute of Agricultural and Nutritional Sciences, Martin Luther University Halle-Wittenberg, Von-Danckelmann-Platz 2, 06120 Halle (Saale), Germany

phone: +49 345 5522707; fax: +49 345 5527124

a

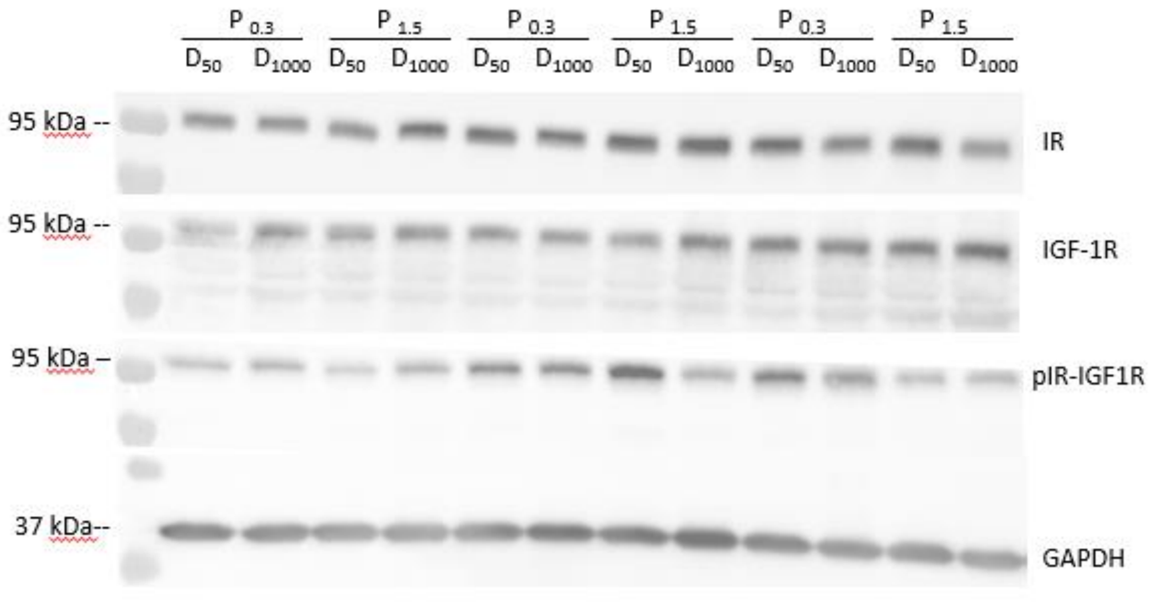

b

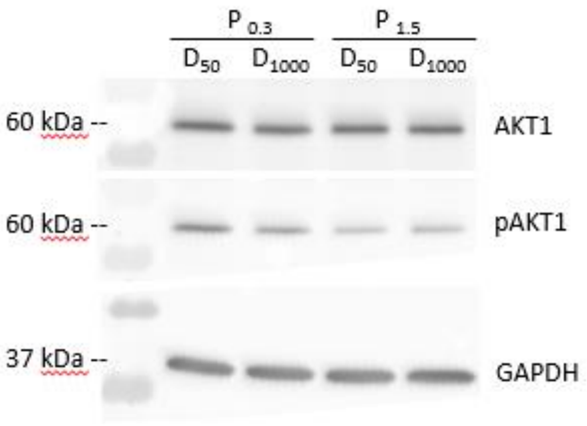

Supplementary Figure S1. The whole uncropped images of the original western blots of (**a**) IR, (**a**) IGF-1R, (**a**) pIR-IGF1R, (**b**) AKT, and (**b**) pAKT1.
